# Oxidative stress-regulatory role of miR-10b-5p in the diabetic human cornea revealed through integrated multi-omics analysis

**DOI:** 10.1007/s00125-025-06558-5

**Published:** 2025-10-20

**Authors:** Daxian Zha, Joshua Gamez, Shaghaiegh M. Ebrahimi, Yizhou Wang, Nagendra Verma, Adam J. Poe, Seok White, Ruchi Shah, Andrei A. Kramerov, Onkar B. Sawant, Chintda Santiskulvong, Aleksandr B. Stotland, Zhiping P. Wang, Jennifer E. Van Eyk, Alexander V. Ljubimov, Mehrnoosh Saghizadeh

**Affiliations:** 1 Biomedical Sciences, Cedars-Sinai Medical Center, Los Angeles, CA USA; 2 Regenerative Medicine Institute, Cedars-Sinai Medical Center, Los Angeles, CA USA; 3 Department of Computational Biomedicine, Cedars-Sinai Medical Center, Los Angeles, CA USA; 4 Center for Vision and Eye Banking Research, Eversight, Cleveland, OH USA; 5 Genomics Core, Cedars-Sinai Medical Center, Los Angeles, CA USA; 6 Advanced Clinical Biosystems Research Institute, The Smidt Heart Institute, Cedars-Sinai Medical Center, Los Angeles, CA USA; 7 David Geffen School of Medicine, University of California Los Angeles, Los Angeles, CA USA

**Keywords:** Corneal limbal stem cells, Diabetic cornea, *GCLM*, Glutamate-cysteine ligase (GCL), Glutathione (GSH), *LANCL1*, miR-10b-5p, miRNA, Oxidative stress, Proteomics, Transcriptomics

## Abstract

**Aims/hypothesis:**

Prominent features of diabetic corneal disease are oxidative stress, neuropathy and epitheliopathy including delayed wound healing and dysfunction of limbal epithelial stem cells. We hypothesised that regulatory miRNAs altered in the diabetic cornea, such as miR-10b-5p, may be responsible for these abnormalities. We aimed to understand the molecular impact of miR-10b-5p increase in human diabetic vs non-diabetic limbal epithelial cells (LECs) enriched in limbal epithelial stem cells by identifying its target genes and proteins and testing it as a potential therapy for inhibiting oxidative stress in diabetic corneas.

**Methods:**

LECs were isolated from diabetic and non-diabetic human autopsy corneas. Telomerase-immortalised human corneal epithelial cells (HCECs), primary LECs and ex vivo organ-cultured corneas were transfected with 50 nmol/l hsa-miR-10b-5p mimic or miRNA inhibitor or siRNA against *GCLM* (glutamate-cysteine ligase modifier subunit) along with their respective controls using Lipofectamine RNAiMAX. Total RNA was extracted for transcriptomic analysis. Proteins were extracted, digested and quantified using LC-MS/MS proteomics. Oxidative stress was induced using 200 µmol/l hydrogen peroxide (H_2_O_2_) in transfected LECs and/or HCECs post starvation. Cell lysates at 0, 3, 6, 9 and 24 h time points were analysed on western blots. Reactive oxygen species in transfected HCECs were measured using the DCFDA/H2DCFDA–Cellular ROS Assay Kit. Glutathione (GSH) levels were quantified using the GSH-Glo assay from H_2_O_2_-treated LECs. Glutamate-cysteine ligase modifier subunit (GCLM) and lanthionine synthetase C-like protein 1 (LANCL1) protein expression levels were also analysed by immunostaining.

**Results:**

Integrative proteomic and genomic analysis of miR-10b- vs miRNA mimic control-transfected primary LECs identified *GCLM* and *LANCL1* as key miR-10b-5p targets (false discovery rate *p*<0.05), validated by western blot and immunostaining. miR-10b- and siRNA-GCLM-transfected LECs 3 h after H_2_O_2_ treatment showed a significant reduction in glutathione/glutathione disulfide (2GSH/GSSG) ratio and overall GSH levels. Further, miR-10b-5p-transfected HCECs produced higher ROS levels, peaking at 12.67 ± 0.22% at 6 h post H₂O₂ treatment, as compared with 10.41 ± 0.20% in controls. This implicates downregulated LANCL1 in modulating cellular responses to oxidative damage. Both GCLM and LANCL1 were downregulated in ex vivo diabetic corneas, while inhibition of miR-10b-5p significantly restored their expression in diabetic organ-cultured corneas by immunostaining.

**Conclusions/interpretation:**

Our findings suggest that diabetes-overexpressed miR-10b disrupts redox balance by targeting *GCLM* and *LANCL1*, which potentially leads to increased oxidative stress and cellular vulnerability in diabetic corneas. Inhibiting miR-10b-5p restored antioxidant defences, suggesting a potential therapeutic strategy to mitigate oxidative stress and normalise corneal health in individuals with diabetes and preserve vision.

**Graphical Abstract:**

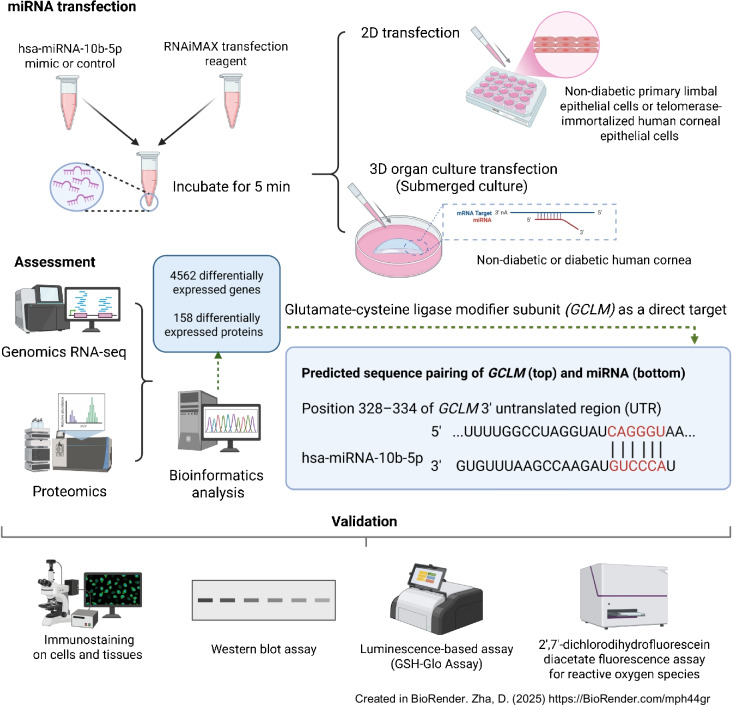

**Supplementary Information:**

The online version contains peer-reviewed but unedited supplementary material available at 10.1007/s00125-025-06558-5.


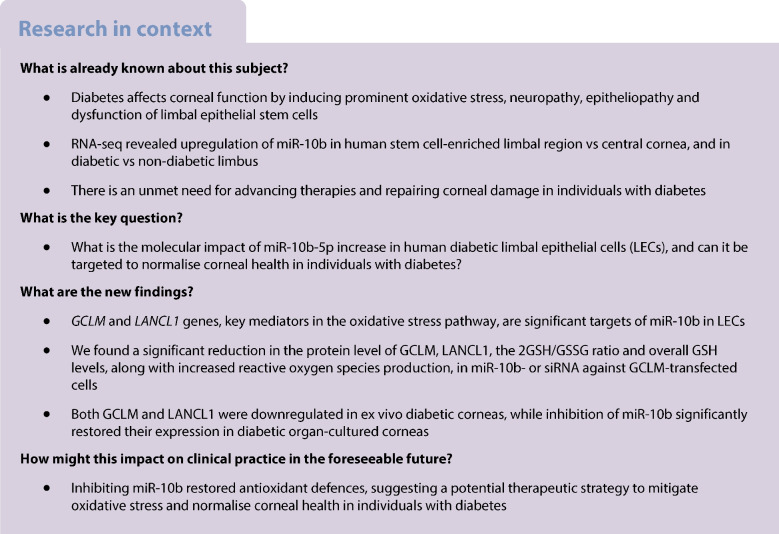


## Introduction

Ocular diseases and injuries affect over 1.1 billion people globally, and corneal health remains critical to maintaining visual function [1]. The cornea, the eye’s transparent front layer, plays a critical role in the visual system due to its exceptional refractive capabilities. The cornea’s outermost layer, the epithelium, undergoes continuous renewal driven by limbal epithelial stem cells (LESCs) residing at the corneoscleral junction, the limbus [2]. LESCs possess remarkable self-renewal potential, generating transit amplifying (TA) cells that migrate towards the central cornea while differentiating to regenerate the stratified epithelial layer [3]. This dynamic process is vital for maintaining corneal epithelial homeostasis and transparency and protecting the eye from environmental insults [4].

Diabetes mellitus is a chronic disease that affects approximately 500 million people worldwide and leads to hyperglycaemia and associated complications due to impaired insulin production and/or function [5]. In individuals with diabetes, corneal health is negatively affected by cellular signalling disruptions, reduced LESC function and compromised TA cell activity, leading to impaired epithelial wound healing and slow regeneration, increased susceptibility to infections, chronic corneal ulcers and ultimately altered vision [6]. Individuals with diabetes exhibit diminished resilience against oxidative stress, a key factor in maintaining epithelial homeostasis [7]. Hyperglycaemia leads to increased production of reactive oxygen species (ROS) and weakens antioxidant defence systems, exacerbating cellular damage and hindering the healing process [8].

miRNAs are small, evolutionarily conserved noncoding RNAs that regulate gene expression post transcriptionally by binding to complementary sequences on the 3′ untranslated region (UTR) of target mRNAs, leading to gene silencing through either mRNA degradation or inhibition of translation [9]. miRNAs have important dynamic roles in regulating key cellular processes including differentiation, proliferation and migration, and have a unique capacity to simultaneously target multiple genes, unlike traditional inhibitors [10, 11]. Recent findings also emphasise miRNAs’ critical functions in stem cell and tissue development, differentiation and maintenance [12, 13]. Previously, we demonstrated that diabetic corneal epithelium exhibits significantly reduced expression of several putative LESC markers, resulting in impaired corneal epithelial wound healing [14]. High-throughput deep sequencing revealed miRNAs, such as miR-10b-5p (miR-10b), validated by real-time quantitative reverse transcription PCR (qRT-PCR) and in situ hybridisation [15], in limbal and central corneas from non-diabetic individuals that were differentially expressed (DE) in type 1 and type 2 diabetes. miR-10b was upregulated in the diabetic limbus, especially in type 1 diabetes [15]. miR-10b overexpression was associated with downregulation of Dickkopf-1 (DKK1), a Wnt signalling inhibitor, and Glycogen synthase kinase-3 beta (GSK-3β), indicating its potential regulatory role in LESC proliferation and differentiation into TA cells, and in diabetic corneal complications [15].

In the present study, we examined in more detail the regulatory role of miR-10b in the limbal epithelium by exploring its potential targets in miR-10b-overexpressed limbal epithelial cells (LECs) using integrated transcriptomic and proteomic analyses. We uncovered a large number of miR-10b target genes in different pathways at the mRNA and protein levels, such as glutamate-cysteine ligase modifier subunit (*GCLM*), a key mediator in the glutathione (GSH) metabolic pathway, which plays a crucial role in response to oxidative stress. Although diabetic corneas experience elevated oxidative stress, the specific role of miR-10b as a molecular regulator in this context remains insufficiently understood. To further elucidate the functional relevance of miR-10b in diabetic corneal pathology, we focused on miR-10b target genes that significantly impact antioxidant defences. Our multi-omics data have documented *GCLM* and lanthionine synthetase C-like protein 1 (*LANCL1*) as potential targets of miR-10b in LECs that can protect cells from oxidative stress. A deeper understanding of miR-10b’s corneal regulatory network could facilitate the development of targeted therapies aimed at restoring oxidative balance and improving corneal integrity in individuals with diabetes.

## Methods

### Human tissues

Deidentified human corneas from donors with similar ages (electronic supplementary material [ESM] Table 1) irrespective of race or ethnicity from 21 non-diabetic (14 male and seven female, 65 ± 13 years) and ten diabetic (five male and five female, 69 ± 5 years) deceased donors were obtained within 24–48 h after death from the National Disease Research Interchange (NDRI, Philadelphia, PA, USA) or Eversight (Ann Arbor, MI; Chicago, IL; Cleveland, OH; and Clark, NJ). The study complied with the Declaration of Helsinki and was approved by Cedars-Sinai Medical Center institutional review board (IRB) (protocol Pro00019393).

### Primary LEC isolation and cell culture and organ culture maintenance

LECs were isolated from the limbus of diabetic and non-diabetic autopsy corneas as we previously documented [16] (ESM Methods).

### Transfection of human primary LECs, HCECs and organ-cultured corneas

Telomerase-immortalised diploid and non-tumourigenic corneal epithelial cell line (human corneal epithelial cells, HCECs) was obtained from D. Dimitrijevich (University of North Texas System) [17, 18]. Human primary LECs, HCECs and non-diabetic and diabetic organ-cultured corneas were transfected with 50 nmol/l hsa-miR-10b-5p mimic (miR-10b) or negative control (miRNA mimic control, miR-MC), using Lipofectamine RNAiMAX, or with 50 nmol/l siRNA-glutamate-cysteine ligase modifier subunit (GCLM) targeting genomic sequence and negative control using DharmaFECT transfection reagents (ESM Methods).

### Oxidative stress induction

To induce oxidative stress in primary LECs and HCECs following the transfection recovery period, the cells were starved for 3 h in EpiLife medium, followed by treatment with 200 µmol/l hydrogen peroxide (H_2_O_2_; Fisher Science Education, Waltham, MA, USA). The cells were maintained at 37°C with 5% CO_2_ throughout the procedure.

### Total RNA isolation, library preparation and next generation RNA-seq

Total RNA was isolated using the PureLink RNA Mini Kit after TRIzol lysis (Thermo Fisher Scientific, Waltham, MA, USA) from non-diabetic (*n*=8) human corneas following the manufacturer’s guidelines. Total RNA was subjected to library preparation and sequencing (ESM Methods).

### Protein extraction and LC-MS/MS analysis and data acquisition and analysis

Primary human LECs (*n*=6) were transfected with miR-10b and its respective control (miR-MC) for 48 h and allowed to recover for a further 24 h before processing. The cell lysates were prepared for proteomics analysis as previously described [19] (ESM Methods).

### Immunostaining and western blot analysis

#### Immunostaining

Cultured primary LECs or 5-µm-thick transverse corneal cryostat sections were fixed using 10% formalin and 1% formalin, respectively, and immunostained (ESM Table 2) as previously documented [20] (ESM Methods).

#### Western blot analysis

Primary LECs and HCECs were transfected with hsa-miR-10b or miR-MC. For HCECs, lysates were collected at the 0, 3, 6, 9 and 24 h time points after H_2_O_2_ oxidation and subjected to western blot analysis (ESM Methods).

### GSH-Glo GSH assay

Primary LECs were seeded into 96-well white opaque plates at 2500 cells per well and transfected with hsa-miR-10b, miR-MC, siRNA-GCLM and its negative control as described above. The cells were exposed to oxidative stress with H_2_O_2_ treatment for 6 h. GSH levels were quantified using the GSH-Glo GSH assay (Promega Corporation, WI, USA) according to the manufacturer’s protocol (ESM Methods).

### DCFDA/H2DCFDA–Cellular ROS Assay

HCECs were seeded into a 96-well plate (*n*=6) and a 48-well plate (*n*=3) at 2000 cells per well and 8000 cells per well, respectively, in culture media. HCECs were evaluated after the oxidative stress protocol at the 0, 3, 6, 9 and 24 h time points. ROS were measured (ESM Methods).

### Statistical analysis

Statistical analysis was performed using GraphPad Prism version 9.2.0 (GraphPad Software, San Diego, CA, USA) by Student’s *t* test for two-group or ANOVA for multiple-group comparisons with *p*<0.05 considered significant. Each experiment was performed in triplicate using samples from at least three different donors. No randomisation or masking was carried out, and no data or samples were excluded from reporting. Values were expressed as mean ± SEM.

## Results

### Transcriptomic analysis of miR-10b-transfected LECs

**Fig. 1:**
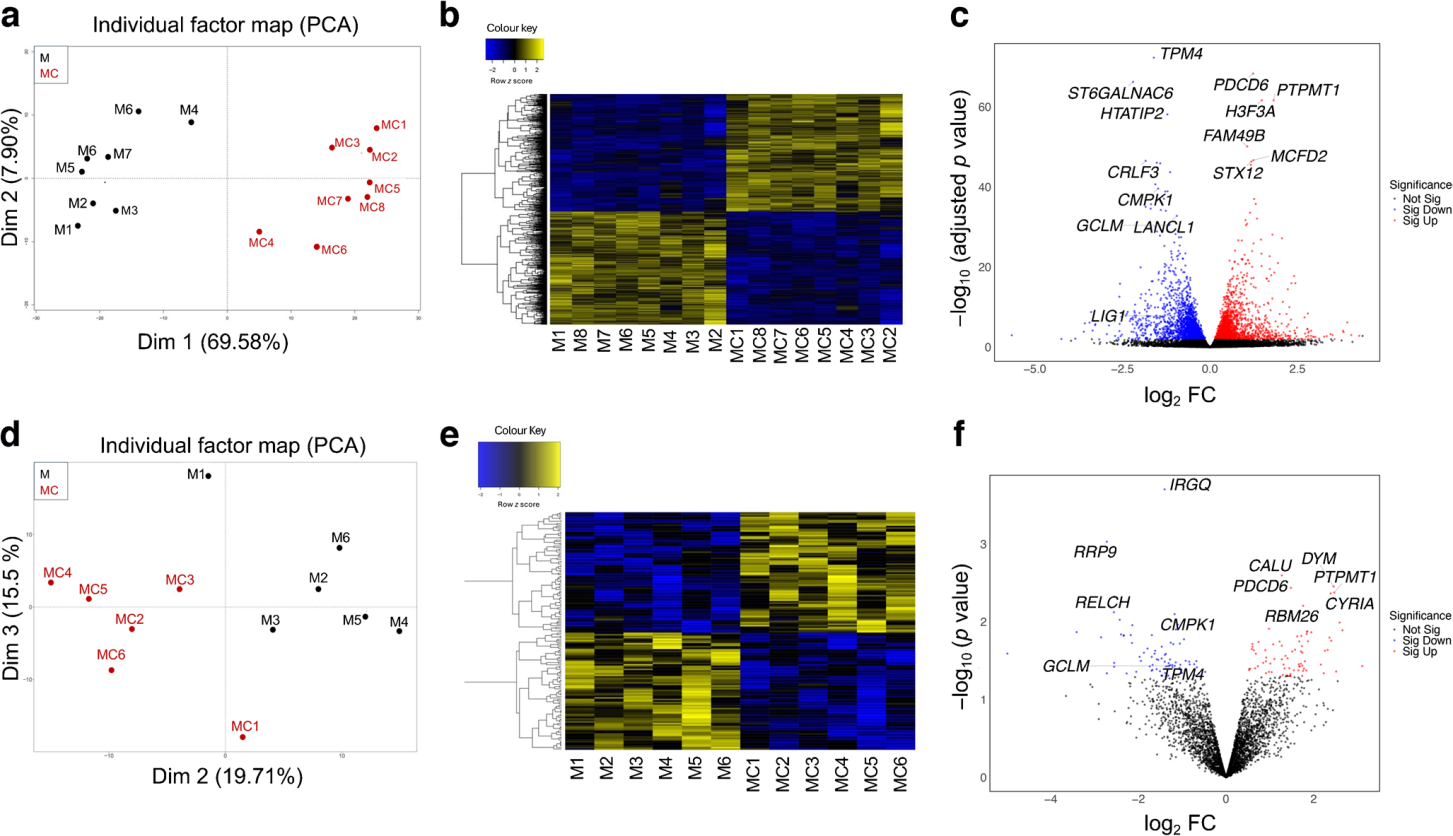
Transcriptomics and proteomics analyses of miR-10b-transfected LECs. (**a**) Unsupervised PCA of genes across all samples reveals two distinct clusters, expressed in miR-10b (black) or miR-MC (red), including individual cases of miR-10b and miR-MC. (**b**) Two-way hierarchical clustering plots were used to generate a heatmap of 4562 DEGs, *p* value <0.05 (2310 were downregulated and 2252 were upregulated) in miR-10b vs miR-MC samples. (**c**) Volcano plot representing the DEGs in LECs transfected with miR-10b vs miR-MC. The *y-*axis represents the mean expression value of −log_10_ (adjusted *p* value), and the *x-*axis represents the log_2_ FC between the experimental groups. Genes that are identified as significantly upregulated are coloured in red and significantly downregulated in blue. The top DEGs of interest are labelled on the volcano plot. (**d**) Unsupervised PCA of proteins across all samples shows two distinct clusters expressed in M (black) or MC (red), including individual cases of M and MC. (**e**) Two-way hierarchical clustering of 158 DEPs, *p*<0.05 (80 were downregulated and 78 were upregulated), indicates altered expression profile in M vs MC samples. (**f**) Volcano plot representing the DEPs in LECs transfected with miR-10b vs miR-MC. The *y-*axis represents the mean expression value of −log_10_ (*p* value), and the *x-*axis represents the log_2_ FC between the experimental groups. Proteins that are identified as significantly upregulated are coloured in red and significantly downregulated in blue. The top DEPs of interest are labelled on the volcano plot. Dim, dimension; M, miR-10b; MC, miR-MC; Not sig, not significant; Sig down, significantly downregulated; Sig up, significantly upregulated

To identify miR-10b target genes, primary human LECs from individual donors (*n*=8) were transfected with miR-10b and its control, miR-MC. Total RNA was isolated 72 h post transfection and significant upregulation of miR-10b- vs miR-MC-transfected LECs was confirmed as shown previously [15]. Principal component analysis (PCA) of RNA-seq data demonstrated substantial transcriptomic differences between LECs transfected with miR-10b vs miR-MC (Fig. 1a). A two-way hierarchical clustering using Euclidean distance and mean linkage (Fig. 1b) showed a clear distinction of 2310 downregulated and 2252 upregulated genes using adjusted *p*<0.05, no fold change (FC) cut off (ESM Table 3). Of these, 383 and 356 differentially expressed genes (DEGs) were downregulated and upregulated, respectively, using false discovery rate (FDR) *p*<0.05 and FC ± 2 in miR-10b- vs miR-MC-transfected LECs. The top interest DEGs are also shown in Fig. 1c as a volcano plot. The volcano plot identified genes with significantly increased (red) or decreased (blue) expression in miR-10b vs miR-MC, including key downregulated candidates such as *GCLM*, *LANCL1*, *TPM4* and *CMPK1*. These genes are implicated in GSH biosynthesis, oxidative damage and mobility, respectively [21–24].

### Proteomic analysis of miR-10b targets in primary LECs

Primary LECs (*n*=6) transfected with miR-10b and miR-MC were analysed by LC-MS/MS and differentially expressed proteins (DEPs) were determined based on proteotypic peptides (peptides comprising amino acid sequence specific to a single protein or isoform). A total of 158 DEPs (80 downregulated, 78 upregulated) were identified with *p*<0.05 (ESM Table 4). PCA plot showed segregation of miR-10b- and miR-MC-transfected samples into two distinct principal groups (Fig. 1d). A two-way hierarchical clustering showed a clear distinction of 78 proteins upregulated and 80 proteins downregulated (*p*<0.05) in miR-10b- vs miR-MC-transfected LECs (Fig. 1e). The volcano plot (Fig. 1f) identified DEPs with miR-10b vs miR-MC with significantly upregulated (red) and downregulated (blue) expression. The top DEPs of interest were highlighted, including key candidates such as cytidine/uridine monophosphate kinase 1 (CMPK1), tropomyosin 4 (TMP4) and heme oxygenase 2 (HMOX2) which exhibited significant downregulation. Among the significantly downregulated proteins, GCLM, essential for GSH biosynthesis, a key antioxidant defence mechanism, was notably reduced. Its downregulation has been linked to increased oxidative stress, rendering cells more susceptible to environmental and metabolic challenges [23].

### Integrated transcriptome and proteome analyses

**Fig. 2:**
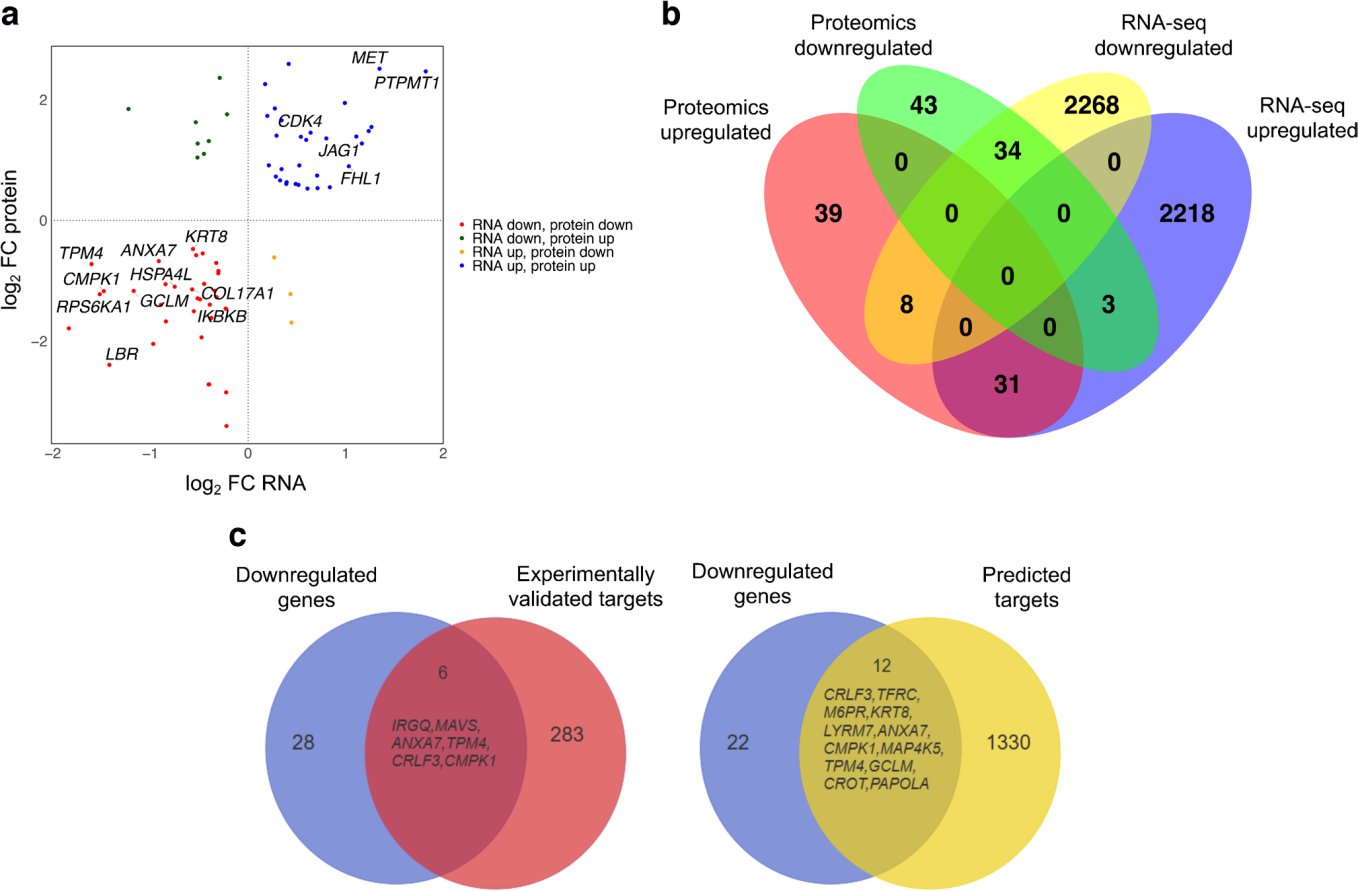
Integrated transcriptomics and proteomics analyses of miR-10b-transfected LECs. (**a**) Overlapped significant DEGs of RNA-seq and proteomics in miR-10b vs miR-MC samples. The *y-*axis represents the log_2_ FC of proteins, and the *x-*axis represents the log_2_ FC of RNAs (genes) DE in LECs transfected with miR-10b vs miR-MC. Upper left and lower right represent the overlapped DE RNA and protein changes in opposite directions; upper left, RNA down- and protein upregulated (green); lower right, RNA up- and protein downregulated (orange). Upper right, both DE RNA and protein are upregulated (blue) and lower left both downregulated (red). (**b**) Venn diagram illustrating the overlap between DE RNAs and proteins in miR-10b- vs miR-MC-transfected LECs. The central overlap (*n*=34) represents entities that were downregulated at both the transcript and protein levels. (**c**) Venn diagrams display the overlap between experimentally validated targets (red circle) and computationally predicted targets (yellow circle) of 34 downregulated miR-10b-5p target genes/proteins (purple) in miR-10b vs miR-MC samples. Of these genes, six out of 34 downregulated genes overlap with 289 experimentally validated miR-10b-5p targets (red). As for computationally predicted targets, 12 out of 34 downregulated genes overlap with 1342 computationally predicted targets (yellow)

Integration of proteomic and genomic analyses of miR-10b- vs miR-MC-transfected primary LECs showed 65 (34 downregulated, 31 upregulated) overlapping DEGs/DEPs, which were present in both RNA-seq and proteomics, FDR *p*<0.05, *p*<0.05, respectively (ESM Table 5). The scatterplot representing transcriptome and proteome analysis of miR-10b- vs miR-MC-transfected LECs (Fig. 2a) revealed significant overlapping DEGs and DEPs downregulated or upregulated in both transcriptomics and proteomics shown in red (lower left) and blue (upper right), respectively. The upper left and lower right represent the overlapped DE RNA and protein changes in opposite directions: upper left, RNA down- and protein upregulated (green); lower right, RNA up- and protein downregulated (orange). The Venn diagram comparison of downregulated miR-10b potential targets by transcriptomics and proteomics revealed 34 overlapping genes/proteins in miR-10b- vs miR-MC-transfected LECs. By contrast, 2268 mRNAs and 43 proteins were downregulated in either transcriptomics or proteomics datasets, respectively (Fig. 2b).

The Venn diagrams (Fig. 2c) depict the overlap between experimentally validated and computationally predicted miR-10b targets among 34 downregulated genes. Specifically, there were 12 genes (Table 1) from the downregulated set overlapping with 1342 computationally predicted targets. Of these, six genes (Table 2) are shared with 289 experimentally validated miR-10b-5p targets. Several genes downregulated by miR-10b, such as *TFRC*, *MAP4K5*, *LYRM7*, *CROT* and ANXA7, are linked to oxidative stress regulation and redox homeostasis. *TFRC* regulates iron intake; its dysregulation can cause oxidative damage via Fenton reactions [24]. *MAP4K5*, a stress-responsive kinase, activates the c-Jun N-terminal kinases (JNK) pathway influencing oxidative stress and apoptosis [25]. *LYRM7* supports mitochondrial electron transport; its dysfunction may increase ROS production [26]. *CROT* is involved in fatty acid metabolism and contributes to redox balance [27], whereas *ANXA7* contributes to membrane repair and calcium signalling, and both intersect with oxidative stress [28]. Together, these genes contribute to cellular resilience or susceptibility to oxidative stress through interconnected metabolic, mitochondrial and signalling pathways, and may be regulated by miR-10b.

**Table 1:** Computationally predicted targets of miR-10b, with an integrated analysis of miR-10b/miR-MC DEGs

Gene	RNA-seq, log_2_FC	Proteomics, log_2_FC
*ANXA7*	−0.914	−0.676
*CRLF3*	−1.835	−1.791
*CMPK1*	−1.478	−1.173
*TPM4*	−1.603	−0.726
*CROT*	−0.477	−1.940
*GCLM*	−1.172	−1.169
*KRT8*	−0.564	−0.475
*LYRM7*	−0.491	−1.309
*M6PR*	−0.375	−1.618
*MAP4K5*	−0.971	−2.048
*PAPOLA*	−0.398	−2.720
*TFRC*	−0.532	−0.577

**Table 2:** Experimentally validated targets of miR-10b, with an integrated analysis of miR-10b/miR-MC DEGs

Gene	RNA-seq, log_2_FC	Proteomics, log_2_FC
*IRGQ*	−0.894	−1.402
*MAVS*	−0.224	−1.468
*ANXA7*	−0.914	−0.676
*CRLF3*	−1.835	−1.791
*CMPK1*	−1.478	−1.173
*TPM4*	−1.603	−0.726

### Functional analysis of DE mRNAs/proteins

**Fig. 3:**
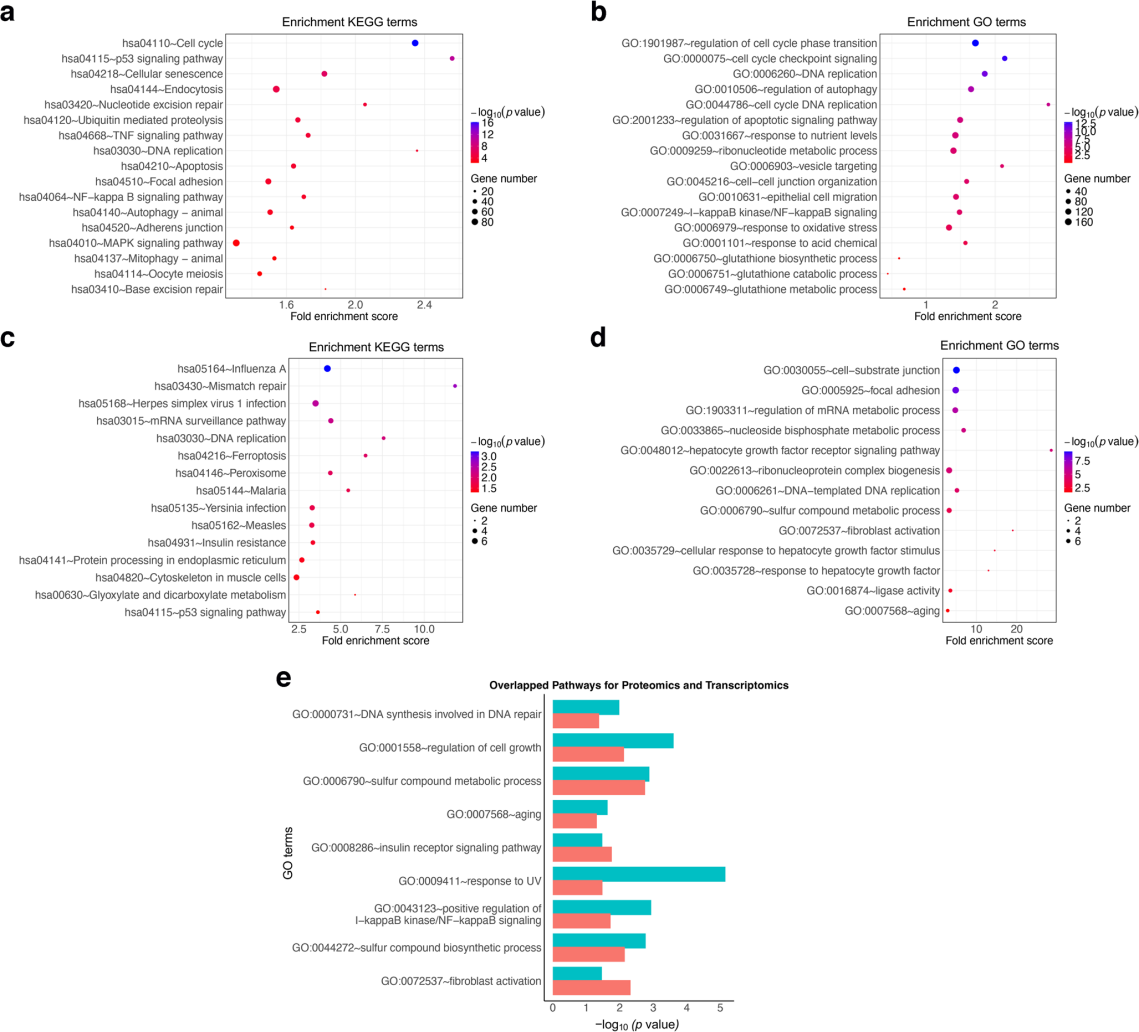
Functional analysis of DE mRNAs/proteins. (**a**) KEGG pathway analysis and (**b**) GO enrichment analysis of DE transcriptomic targets reveal significant pathways that are involved in corneal pathophysiology. (**c**) KEGG pathway analysis and (**d**) GO enrichment analysis of DE proteomic targets reveal significant pathways that are involved in corneal pathophysiology. (**e**) Common functional pathways enriched in DE transcriptomic and proteomic targets in miR-10b- vs miR-MC-transfected LECs reveal the altered key pathways. The *y*-axis represents the GO terms, and the *x-*axis represents the mean expression value of −log_10_ (*p* value) of the overlapped pathways. Proteomic data are shown in red and transcriptomic data are shown in blue

The multi-omics analyses revealed significant alterations in multiple pathways associated with cell cycle, apoptosis, inflammation, oxidative stress and metabolic processes. Kyoto Encyclopedia of Genes and Genomes (KEGG) pathway analysis of transcriptomic data identified significant enrichment in pathways related to cell cycle regulation, DNA replication, cellular senescence, apoptosis, NF-κB signalling and metabolic processes (Fig. 3a). Gene ontology (GO) enrichment analysis of transcriptomic data further confirmed these findings by highlighting significant regulation in cell cycle checkpoint signalling, DNA replication, apoptotic pathways and NF-κB signalling (Fig. 3b). Additionally, transcriptomic data revealed strong enrichment in pathways related to oxidative stress responses and GSH metabolism, indicating an adaptive cellular response to oxidative damage and redox imbalances. Specifically, miR-10b was found to interfere with this protective mechanism by downregulating key genes involved in oxidative stress, such as *GCLM* [23], *LANCL1* [29], *SLC23A2* [30] and *HSPA1A*, a molecular chaperone that helps cells survive under stress [31]. These genes play pivotal roles in maintaining antioxidant defences and modulating stress response [23, 29–32]. Analysis of proteomic data using KEGG pathway enrichment (Fig. 3c) demonstrated significant changes in DNA replication and repair pathways, consistent with transcriptomic findings. Moreover, proteomic data highlighted alterations in insulin resistance pathways, suggesting metabolic regulation. GO pathway enrichment of proteomic data (Fig. 3d) identified significant regulation of multiple metabolic pathways, including sulphur compound metabolism and hepatocyte growth factor receptor signalling. These results suggest that proteomic alterations complement transcriptional changes, reinforcing the biological significance of these pathways.

We further analysed shared pathways between transcriptomic and proteomic datasets (Fig. 3e). Several key pathways, including DNA repair, cell growth regulation and metabolic processes, were significantly enriched in both datasets. Response to ultraviolet radiation and positive regulation of NF-κB signalling were predominantly enriched in transcriptomics, whereas proteomics highlighted essential metabolic processes. Downregulation of *GCLM* in these overlapped pathways, including sulphur compound metabolic and biosynthetic processes, ageing and fibroblast activation pathways associated with free radical formation, further revealed the regulatory role of miR-10b in oxidative stress responses by targeting *GCLM*.

### miR-10b suppresses the expression of GCLM, but not GCLC, the GCL subunit

**Fig. 4:**
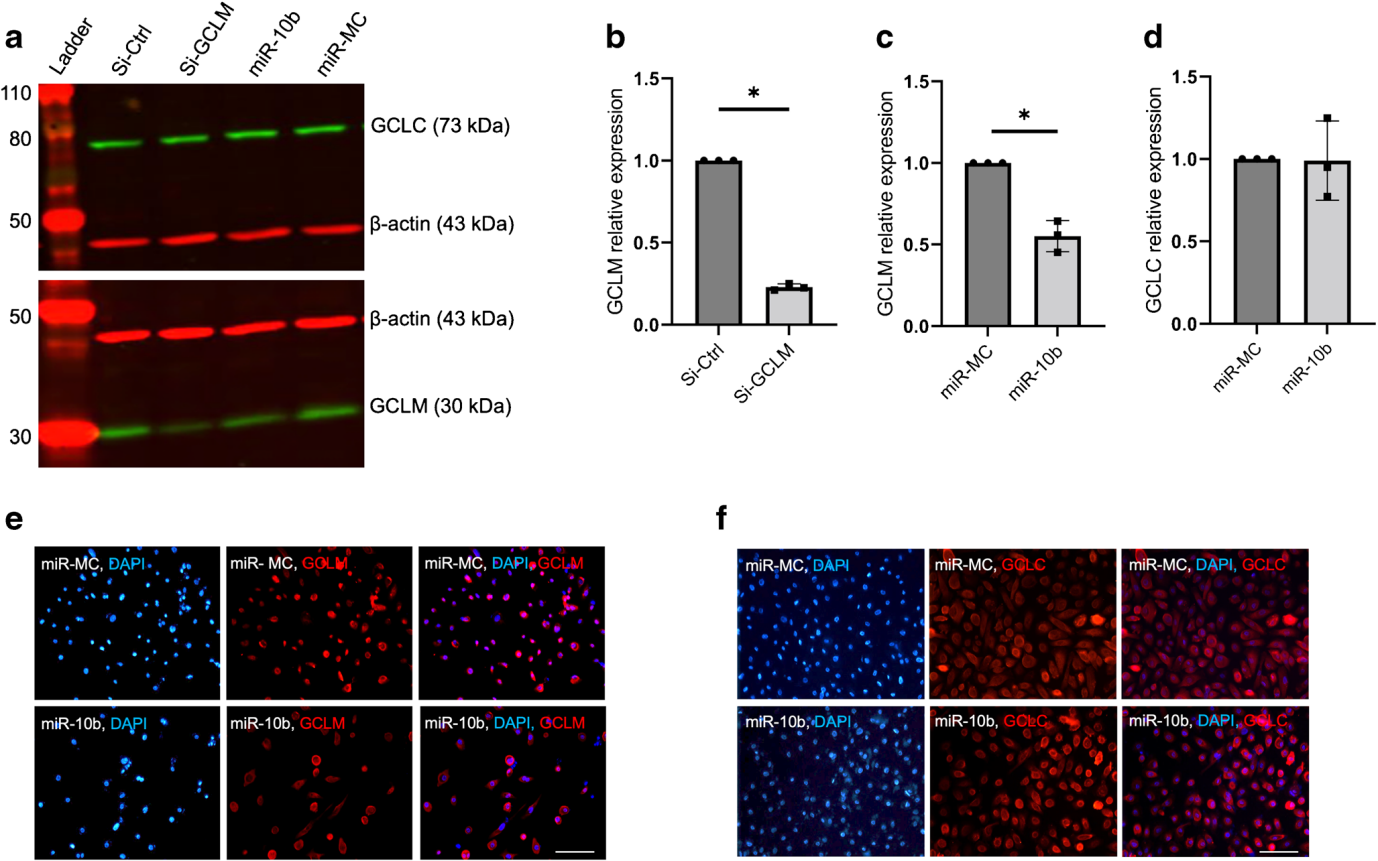
Identification and validation of miR-10b targeting GCLM in human primary LECs. miR-10b comparable to GCLM-specific siRNA downregulates the expression of GCLM but not GCLC. (**a**) Total extracted protein from primary human LECs transfected with 50 nmol/l miR-10b or siRNA-GCLM (Si-GCLM) and their respective controls, miR-MC and Si-Ctrl, was separated on gradient SDS-PAGE gels, transferred to nitrocellulose and probed with primary antibodies to GCLM and GCLC. Antibody to β-actin was used as loading control and for quantification. (**b**–**d**) Protein levels were quantified using Image Studio software (version 5.2) on Odyssey Clx System (LI-COR Biotechnology), and bar graphs were generated using GraphPad Prism. (**b**) GCLM-specific siRNA (Si-GCLM) vs control (Si-Ctrl) downregulates the expression of GCLM. (**c**) miR-10b vs miR-MC downregulates the expression of GCLM. (**d**) There is not a significant change in GCLC protein level in miR-10b- vs miR-MC-transfected cells. Bar graphs represent average ± SEM of pooled values (*n*=3) of densitometric scans compared with control values by paired two-tailed *t* test. *p<0.05. (**e**) Fluorescence microscopy of fixed and immunostained primary LECs transfected with miR-10b showed decreased expression of GCLM (red) compared with miR-MC. Cell nuclei were immunostained with DAPI (blue). (**f**) Fluorescence imaging of primary LECs transfected with miR-10b revealed no significant difference in GCLC expression (red) compared with miR-MC, as assessed by immunostaining. Cell nuclei were counterstained with DAPI (blue). The same exposure time was used for each set of compared immunostained sections; the pictures are representative of three independent experiments of each transfected primary LEC (*n*=3). Scale bars, 20 μm

Integrated multi-omics analyses demonstrated significant downregulation of GCLM following miR-10b transfection. GCLM functions as the regulatory subunit of glutamate-cysteine ligase (GCL), the rate-limiting enzyme in the GSH synthesis pathway. GCL also includes a catalytic subunit, glutamate-cysteine ligase catalytic subunit (GCLC), responsible for initiating GSH synthesis [33]. Primary LECs treated with miR-10b showed significant downregulation of GCLM by western blot compared with negative miR-MC control (Fig. 4a, b). Accordingly, there was a significant decrease in GCLM expression in siRNA-*GCLM* treated primary LECs vs siRNA-negative control (Fig. 4a, c). However, there was no significant change in GCLC expression in miR-10b-treated cells compared with miR-MC (Fig. 4a, d). Evidence supports that GCLM and GCLC are expressed independently of each other as shown by the selective knockdown effect of miR-10b and siRNA. Immunostaining of primary LECs treated with miR-10b showed a similar decrease in GCLM compared with miR-MC control (Fig. 4e). Again, there was no significant difference in GCLC expression compared with the miR-MC (Fig. 4f). These data validate the expression of GCLM and GCLC within primary LECs and support miR-10b suppression of GCLM expression.

### miR-10b decreases GCLM expression, GSH level and 2GSH/GSSG ratio

**Fig. 5:**
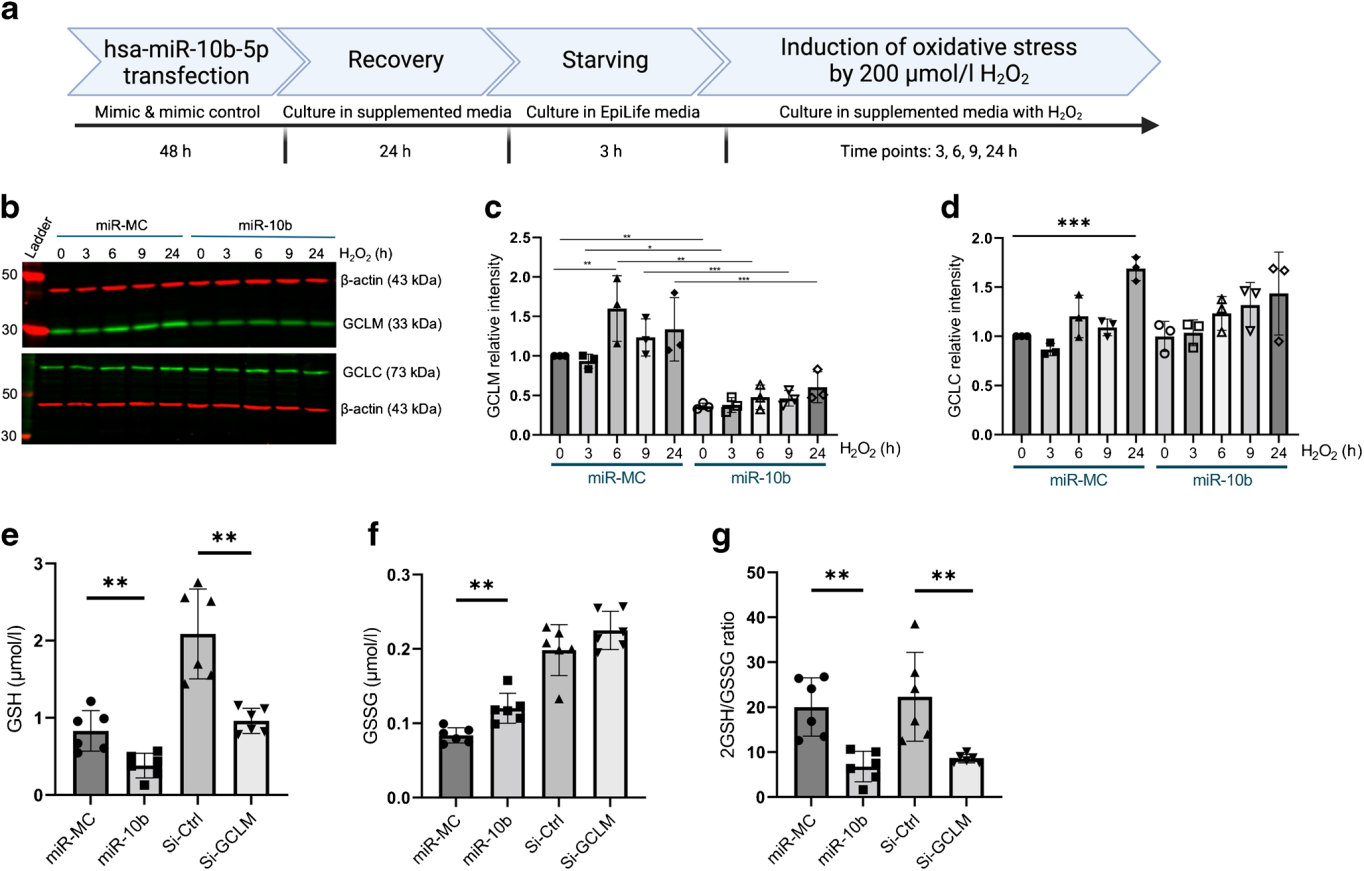
miR-10b decreases GCLM expression, GSH level and 2GSH/GSSG ratio. (**a**) Schematic illustration of the experimental timeline for evaluating hsa-miR-10b-5p effects. HCECs or LECs were transfected with miR-10b-5p or control for 48 h, followed by 24 h recovery, 3 h starvation and oxidative stress induction using 200 µmol/l H₂O₂, with evaluations at 3, 6, 9 and 24 h post treatment. (**b**) GCLM downregulation is maintained after H_2_O_2_ oxidative stress treatment of miR-10b- vs miR-MC-transfected HCECs. Total extracted proteins from HCECs were separated on gradient SDS-PAGE gels, transferred to nitrocellulose and probed with primary antibodies to GCLM and GCLC (ESM Table 2). Antibody to β-actin was used as loading control and for semi-quantification. (**c**) GCLM protein levels across all experimental groups were quantified using Image Studio software, and bar graph generated using GraphPad Prism. (**d**) GCLC protein levels across all experimental groups were quantified using Image Studio software, and bar graph generated using GraphPad Prism. Bar graphs represent average ± SEM of pooled values (*n*=3) of densitometric scans compared with negative control values by one-way ANOVA. *p<0.05, **p<0.01, ***p<0.001. (**e**–**g**) GSH luminescence-based assay. Transfected primary LECs with siRNA-GCLM (Si-GCLM) or miR-10b or respective negative controls, Si-Ctrl and miR-MC, were treated with 200 µmol/l H_2_O_2_ after 3 h starvation for 6 h prior to quantification. (**e**) Reduced (GSH), (**f**) oxidised (GSSG) and (**g**) 2GSH/GSSG ratio forms of GSH were measured and quantified using a LUMIstar Omega luminescence plate reader. Total GSH, total GSSG and 2GSH/GSSG ratio levels were determined as indicated in the Methods. Bar graphs represent average ± SEM of pooled values (*n*=6) compared with negative control values by one-way ANOVA. **p<0.01

GCL subunits are influenced by cellular stimuli that include oxidative stress, as GSH is an important antioxidant protecting cells from stress [33, 34]. To evaluate whether GCLM downregulation was also evident in the presence of oxidative stimuli, we treated HCECs with H_2_O_2_ for different time periods in the presence or absence of exogenous miR-10b (Fig. 5a). miR-10b overexpression was associated with a repressive response to the expected upregulation of GCLM in control (miR-MC)-transfected cells (Fig. 5b, c). In control samples, significant GCLM expression was observed from 0 to 24 h after H_2_O_2_ treatment with a peak expression at 6 h compared with miR-10b-overexpressing cells. And there was no significant change in GCLC expression level in miR-10b- vs miR-MC-transfected cells at different time points of H_2_O_2_ treatment. However, as expected, a significant increase in GCLC expression occurred at 24 h of H_2_O_2_ treatment in control-transfected cells (Fig. 5b, d).

To test miR-10b-mediated GCLM downregulation in the final product of the biosynthetic pathway, we measured GSH and glutathione disulphide (GSSG) levels in stressed primary LECs using luminescence assay. Both reduced (GSH) and oxidised (GSSG) forms of GSH were measured and quantified 3 h after H_2_O_2_ treatment of transfected LECs with miR-10b or siRNA-GCLM as a positive control or their respective controls, using LUMIstar Omega luminescence plate reader. LECs treated with both miR-10b and siRNA-GCLM showed significant reduction in GSH levels vs respective controls (Fig. 5e). GSSG levels were significantly increased in miR-10b-transfected LECs vs the respective control, but not siRNA-GCLM (Fig. 5f). However, both miR-10b- and siRNA-GCLM-transfected cells showed a significant reduction in 2GSH/GSSG ratio and overall GSH levels (Fig. 5g).

### miR-10b increases ROS and downregulates LANCL1

**Fig. 6:**
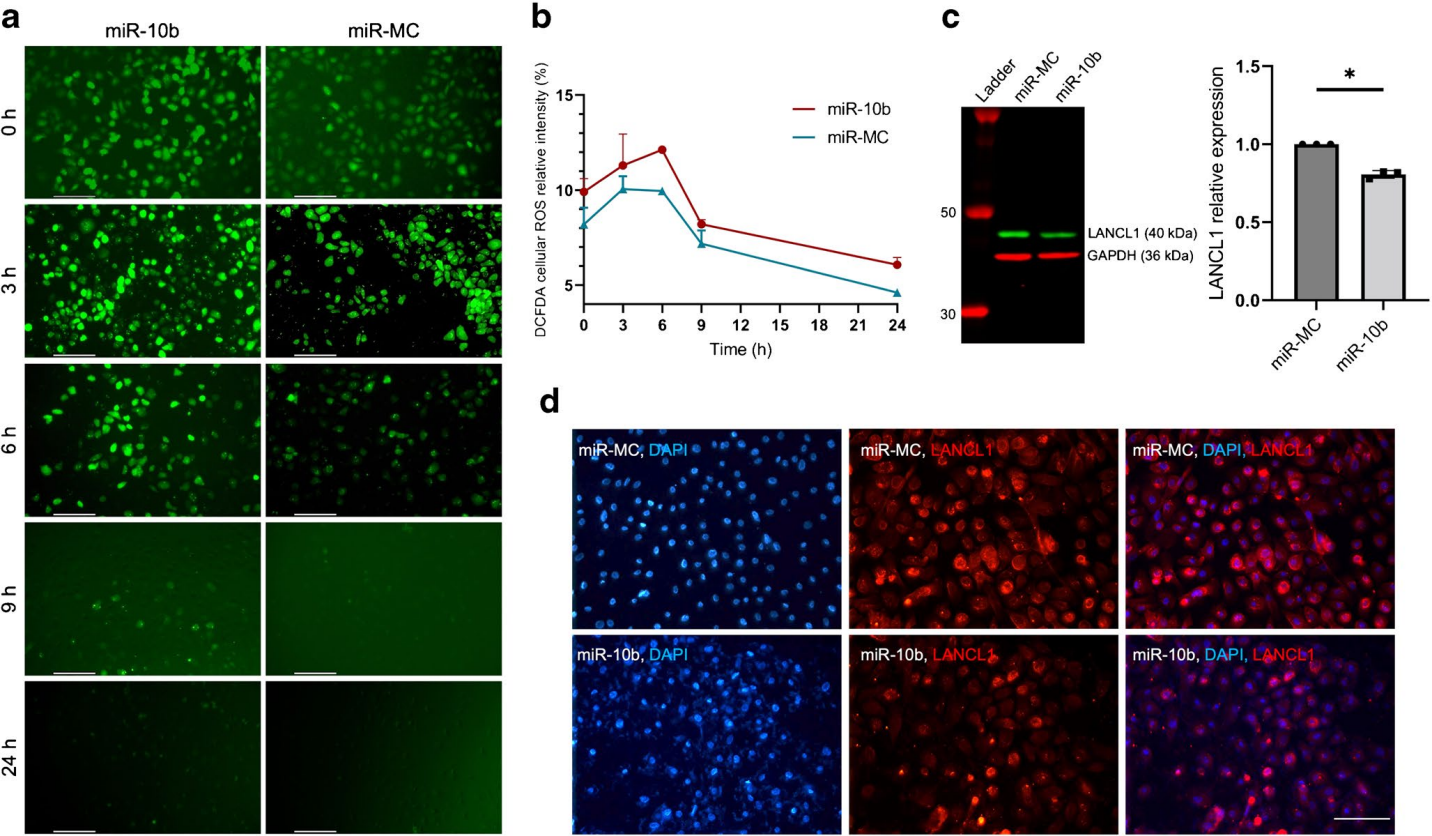
miR-10b increases ROS and downregulates LANCL1. (**a**) Fluorescence microscopy images of live miR-10b- vs miR-MC-transfected HCECs treated with 200 µmol/l H_2_O_2_ at 0, 3, 6, 9 and 24 h time points. Cells were stained with DCFDA solution (green), which is an indicator for ROS. The same exposure time was used for each set of stained sample sections; the pictures are representative of three independent experiments of each transfected HCEC (*n*=3). Scale bars, 130 μm. (**b**) Line graph representing HCEC quantified ROS levels using a fluorescence plate reader at excitation/emission 485/535 nm after transfection with miR-10b and miR-MC and oxidation with 200 µmol/l H_2_O_2_. ROS levels were measured at 0, 3, 6, 9 and 24 h time points. Quantification of ROS levels was normalised to the *tert*-butyl hydroperoxide (TBHP) positive control, and the relative intensity percentage was calculated. Graph represents average ± SEM of pooled values (*n*=3) of fluorescence scans. miR-10b in red, miR-MC in blue. (**c**) Western blot analysis of primary human LECs transfected with 50 nmol/l miR-10b or miR-MC and probed with primary antibodies to LANCL1. Antibody to GAPDH was used as loading control and for quantification. Protein levels were quantified using Image Studio software, and bar graphs were generated using GraphPad Prism. Bar graphs represent average ± SEM of pooled values (*n*=3) of densitometric scans compared with control values by paired two-tailed *t* test. *p<0.05. (**d**) Primary LECs transfected with miR-10b showed decreased expression of LANCL1 (red) compared with the control by immunostaining. Cell nuclei were immunostained with DAPI (blue). The same exposure time was used for each set of compared immunostained sections; the pictures are representative of three independent experiments of each transfected primary LEC (*n*=3). Scale bar, 20 μm

To evaluate oxidative stress levels, a dichlorodihydrofluorescein diacetate (DCFDA) Cellular ROS Assay was performed on miR-10b-transfected HCECs treated with H_2_O_2_. To assess ROS expression and localisation, fluorescence images were captured at different time points (Fig. 6a). The data suggest a higher proportion of ROS in miR-10b- vs miR-MC-transfected HCECs as evidenced by enhanced fluorescence intensity. A subsequent decline in ROS levels was observed in both groups by 24 h (Fig. 6a). miR-10b-transfected HCECs produced higher ROS levels, peaking at 12.67 ± 0.22% at 6 h post H₂O₂ treatment, as compared with miR-MC which peaked at 10.41 ± 0.20% (Fig. 6b). Western blot (Fig. 6c) and immunostaining (Fig. 6d) of miR-10b- vs miR-MC-transfected LECs confirmed the transcriptomics result of downregulation of lanthionine synthetase C-like protein 1 (LANCL1), which is implicated in modulating cellular responses to oxidative damage and maintaining ROS homeostasis [35].

### GCLM and LANCL1 expression in ex vivo and organ-cultured corneas

**Fig. 7:**
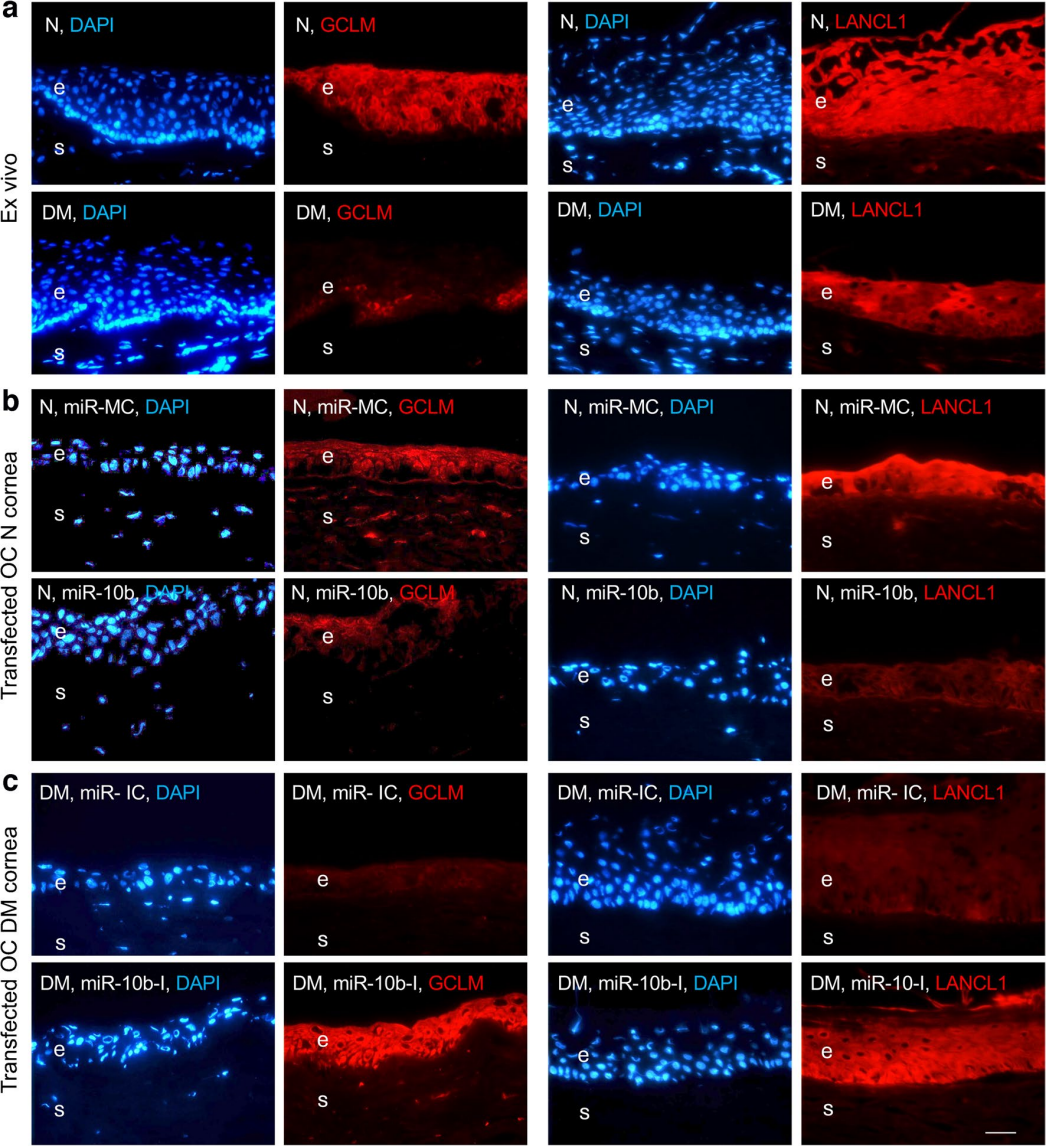
GCLM and LANCL1 expression in diabetic and non-diabetic human ex vivo and organ-cultured corneas. (**a**) GCLM and LANCL1 expression is downregulated in diabetic (DM) vs non-diabetic (N) ex vivo human corneas. (**b**) Both GCLM expression and LANCL1 expression are downregulated in miR-10b- vs miR-MC-transfected N organ-cultured corneas. (**c**) miR-10b inhibitor (miR-10b-I) normalises GCLM and LANCL1 expression in human DM organ-cultured corneas. Both GCLM expression and LANCL1 expression are upregulated in miR-10b- vs inhibitor control (miR-IC)-transfected DM organ-cultured corneas. The same exposure time was used for each set of compared immunostained sections; the pictures are representative of three independent experiments of each sample (*n*=3). Scale bar, 20 μm. e, epithelium; OC, organ-cultured; s, stroma

To confirm the effects of miR-10b on the expression of GCLM and LANCL1 in the human cornea, immunostaining of ex vivo and organ-cultured human corneas was performed. GCLM and LANCL1 were downregulated in diabetic vs non-diabetic ex vivo corneas (Fig. 7a). Similarly, non-diabetic organ-cultured corneas transfected with miR-10b showed downregulation of GCLM and LANCL1 expression compared with miR-MC-transfected organ-cultured corneas (Fig. 7b). GCLM and LANCL1 expression was detected in both limbal and central corneal epithelium, whereas GCLM but not LANCL1 showed positive expression in stromal cells. We attempted to normalise these alterations in diabetic organ-cultured corneas by inhibiting miR-10b. This led to markedly enhanced GCLM and LANCL1 expression compared with miR inhibitor control (miR-IC)-transfected diabetic organ-cultured corneas (Fig. 7c).

These data support that GCL subunits are influenced by oxidative stimuli via H_2_O_2_ in a time-dependent manner, suggesting an inverse regulation of GCLM and miR-10b-5p expression when oxidative stress is present.

## Discussion

Previously, we identified miRNA expression profiles in human diabetic and non-diabetic limbus and found a number of miRNAs such as miR-10b and miR-146a that were upregulated in limbal vs central and in diabetic vs non-diabetic corneas [15]. Using combined transcriptomics and proteomics analysis, we previously provided the first comprehensive molecular insight into miR-146a regulatory roles in corneal epithelium [36]. In the present study, we used the same strategy to provide the first comprehensive study of the regulatory role of miR-10b in diabetic vs non-diabetic corneal epithelium. Our multi-omics analyses indicated that miR-10b significantly downregulates the expression of numerous genes and proteins. The integrated analysis revealed 65 overlaps in mRNA/protein targets (FDR *p*<0.05) in miR-10b- vs miR-MC-transfected LECs. Out of 65 DEGs, 34 including *GCLM* were downregulated and can be potentially considered as direct targets of miR-10b in LECs. The functional analysis of DE mRNAs and proteins indicated that miR-10b may play a role in modulating the oxidative stress pathway. The GO pathway of transcriptomics and integrated analyses indicated that *GCLM* downregulation (shown by western blot and immunostaining) contributed to multiple pathways, including sulphur compound biosynthetic process, sulphur compound metabolism, cellular response to oxidative stress, fibroblast activation and ageing pathways. Given its extensive involvement in multiple critical pathways, our study concludes that *GCLM* is a direct target of miR-10b, corroborating previous findings on its pivotal role in GSH biosynthesis and antioxidant defence mechanisms [37, 38].

Oxidative stress plays a pivotal role in the pathogenesis of keratoconus [39] and in diabetic corneal dysfunction [40–42]. GCLM functions as part of GCL, the rate-limiting enzyme in GSH synthesis, which is vital for neutralising ROS [23]. The KEGG and GO pathway enrichment analyses identified significant disruptions in metabolic, apoptotic and oxidative stress-related pathways, highlighting the multifaceted role of overexpression of miR-10b in corneal epithelial dysfunction. miR-10b's downregulation of *ACSL4*, *SNCA*, *GCLM*, *SLC25A1* and *ACSS2* genes suggests a profound impact on sulphur compound metabolism and biosynthesis, processes crucial for maintaining cellular homeostasis [43–46] (ESM Table 5). Notably, sulphur compound metabolism is critical for maintaining redox balance and regulating oxidative stress [47]. Sulphur amino acid metabolism, particularly via the transmethylation/transsulphuration pathway, supports cellular redox homeostasis by converting homocysteine to cysteine [48]. Sulphur compounds such as hydrogen sulphide (H_2_S) function as gasotransmitters and regulate redox balance by modulating GSH generation and nuclear factor erythroid 2-related factor 2 (NRF2) activity [49]. Cysteine, a precursor of H_2_S, enhances GSH synthesis by reducing cystine into cysteine [50]. Our functional studies show that miR-10b overexpression decreases the 2GSH/GSSG ratio under oxidative stress. Another interesting finding is that the control cells exhibited a significant increase in GCLC expression at 24 h, whereas the miR-10b-transfected cells did not show any significant changes over time. The reason for GCLC's delayed response to GCLM may involve the temporal dynamics of GSH depletion and the subsequent induction of GCLC [51]. The higher expression of GCLM effectively enhances GCL activity more than GCLC alone by forming the holoenzyme complex, which reduces the *K*_m_ and boosts catalytic efficiency without necessitating a corresponding rise in GCLC levels [52]. Collectively, the results suggest that miR-10b overexpression disrupts antioxidant defences, increasing susceptibility to oxidative damage and further compromising corneal integrity in diabetic conditions.

Similarly, LANCL1 plays a crucial role in oxidative stress response, as well as mitochondrial function. LANCL1 has been implicated in cellular redox homeostasis by modulating metabolic pathways and mitigating oxidative stress-induced mitochondrial dysfunction [53]. We show that miR-10b suppresses LANCL1, leading to increased ROS accumulation in HCECs. This observation suggests that miR-10b contributes to oxidative stress by reducing LANCL1-mediated protective mechanisms. Immunostaining and western blot further validated these findings, showing decreased LANCL1 in miR-10b-transfected cells. These results are consistent with prior research demonstrating the protective role of LANCL1 in ROS homeostasis [30, 35, 53].

Our organ culture experiments provided further evidence that miR-10b inhibition restores GCLM and LANCL1 expression, rescuing antioxidant capacity in diabetic corneas. miR-10b could, therefore, serve as a therapeutic target to enhance antioxidant defences and mitigate oxidative stress-related damage in the corneal epithelium.

Despite these significant findings, this study has limitations. Whereas our transcriptomic and proteomic analyses provide strong molecular insights into miR-10b regulation, the impact of miR-10b on progenitor cell properties remains to be established.

miRNAs exert their regulatory effects by targeting different genes in a cell type-specific manner, influencing various cellular functions and disease outcome. For example, miR-203a inhibits p63 in skin but targets Wnt-5a, not p63, in the cornea [54]. Similarly, discrepancies in the role of miR-10b in diabetes [55] may stem from its context-dependent targeting influenced by tissue type, target availability, disease stage and interactions with other miRNAs and feedback loops. These context-dependent regulatory mechanisms underscore the functional complexity of miR-10b in diabetes and the importance of conducting tissue- and condition-specific studies to validate its clinical relevance.

Clinical manifestations of diabetic corneal disease, such as epithelial fragility, nerve dysfunction and impaired epithelial adhesion and wound healing, compromise visual acuity and ocular surface integrity. These changes are largely associated with oxidative stress, driven by chronic hyperglycaemia-induced accumulation of advanced glycation end products (AGEs), overproduction of ROS and impaired antioxidant defences. This is known to lead to cellular damage and dysfunction of the corneal epithelium and nerves [56]. Our study identifies miR-10b as a key regulator in this process, as it downregulates genes involved in oxidative stress and cellular survival pathways. In addition, its targeting of key genes such as *KLF4* (maintaining epithelial barrier integrity), *DKK1* (preserving the limbal stem cell phenotype via *Wnt* inhibition) and *PAX6* (supporting corneal cell identity and adhesion), as previously shown [15], highlights its contribution to diabetic corneal pathology. In summary, our findings highlight the critical role of miRNAs in regulating LEC homeostasis and offer novel insights into the function of miR-10b-5p in corneal epithelial cells. This study provides the first evidence of the regulatory roles of miR-10b in the corneal oxidative stress pathway and suggests that inhibiting miR-10b may present a promising therapeutic strategy to restore corneal health in individuals with diabetes.

## Supplementary Information

Below is the link to the electronic supplementary material.

ESM (PDF 211 KB)

ESM Tables (XLSX 323 KB)

## Data Availability

All results of this study are included in the manuscript and ESM. Data and resources are available upon request from the corresponding author. The RNA-seq dataset is available from the Gene Expression Omnibus (GEO) using the accession number GSE292721, and the proteomics data are available on MassIVE (ID: MSV000097328).

## Abbreviations

DCFDA: Dichlorodihydrofluorescein diacetate
DE: Differentially expressed
DEG: Differentially expressed gene
DEP: Differentially expressed protein
FC: Fold change
FDR: False discovery rate
GCL: Glutamate-cysteine ligase
GCLC: Glutamate-cysteine ligase catalytic subunit
GCLM: Glutamate-cysteine ligase modifier subunit
GO: Gene ontology
GSH: Glutathione
GSSG: Glutathione disulfide
HCEC: Human corneal epithelial cell
H_2_O_2_: Hydrogen peroxide
H_2_S: Hydrogen sulphide
KEGG: Kyoto Encyclopedia of Genes and Genomes
LANCL1: Lanthionine synthetase C-like protein 1
LEC: Limbal epithelial cell
LESC: Limbal epithelial stem cell
miR-10b: miR-10b-5p
miR-IC: miRNA inhibitor control
miR-MC: miRNA mimic control
PCA: Principal component analysis
ROS: Reactive oxygen species
TA: Transit amplifying
