# Supplementary material for: Oxidative stress-regulatory role of miR-10b-5p in the diabetic human cornea revealed through integrated multi-omics analysis
Source: Diabetologia. 2025 Oct 20;69(1):198–213. doi: 10.1007/s00125-025-06558-5 (PMC12686106; doi:10.1007/s00125-025-06558-5)
Supplement: Supplementary file 1 — ESM (PDF 211 KB) [file 125_2025_6558_MOESM1_ESM.pdf]

Electronic Supplementary Material (ESM)

ESM Methods

2. Research Design and Methods

2.2. Primary Limbal Epithelial Cell Isolation, Cell Culture & organ-cultured Maintenance.

Epithelial cells were detached by submerging the tissue in dispase II (Roche Life Science, Pleasanton, CA, USA, cat# 04942078001) (2.4 U/mL) at 37°C for 2 h. The detached epithelial cells were further dissociated using 0.25% trypsin–EDTA (Thermo Fisher, Carlsbad, CA, USA, cat# 25200056) for 30 min at 37°C (1). Cells were placed in plates coated with 1X-Hanks' Balanced Salt Solution (FUJIFILM Irvine Scientific, Santa Ana, CA, USA, cat# 9228) containing human fibronectin (Corning, Tewksbury, MA, USA, cat# 354008, 1 mg/cm^2), type IV collagen (Sigma Aldrich, St. Louis, MO, USA, cat# CC076, 1 mg/cm^2), and laminin-521 (Thermo Fisher, cat# A29248, 0.3 mg/cm^2) (1,2). Epithelial cells were cultured in EpiLife medium (Thermo Fisher, cat# MEPI500CA) supplemented with human keratinocyte growth supplement (HKGS) (Thermo Fisher, cat# S0015), N-2 (Thermo Fisher, cat# 17502048), B27 (Thermo Fisher, cat# 17504044), 15 ng/mL of human epithelial growth factor (hEGF) (Sigma Aldrich, cat# E9644), and 1X antibiotic/antimycotic (Thermo Fisher, cat# 15240062) (1, 2). Telomerase-immortalized human corneal epithelial cells (HCEC) obtained from Dr. S. Dan Dimitrijevich were grown in Epilife medium (Thermo Fisher, cat# MEPI500CA) supplemented with HKGS (Thermo Fisher, cat# S0015) and 1X antibiotic/antimycotic (Thermo Fisher, cat #15240062) (3). Corneal organ cultures were established according to the previously described method (4). Corneal organ cultures were maintained in Dulbecco's Modified Eagle's Medium, low glucose (Thermo Fisher, cat # 11885084) supplemented with 1X Insulin-Transferrin- Selenium (Thermo Fisher, cat# 41400045), 1X non-essential amino acids (Thermo Fisher, cat# 11140050), and 1X antibiotic/antimycotic (Thermo Fisher, cat# 15240062).

2.3. Transfection of Human Primary LECs, HCECs and Organ-cultured Corneas

Human primary LECs and HCECs were transfected with hsa-miR-10b-5p mimic (miR10b) or negative mimic control (miR-MC) (Thermo Fisher, cat# 4464066 ASSAY ID: MC11108, cat# 4464058) at a concentration of 50 nM using Lipofectamine RNAiMAX (Thermo Fisher, cat# 13778075). Transfection media were prepared by diluting the reagents in OPTI-MEM ™ (Thermo Fisher, cat# 31985062) and their corresponding cell culture medium per manufacturer's specification. Human primary LEC and HCECs were transfected with siRNA GCLM targeting genomic sequence and negative control (Dharmacon, Lafayette, CO, USA, cat# L-011670-01-0005, cat# D-001810-10-05) at a concentration of 50 nM using DharmaFECT transfection reagent (Dharmacon, cat# T-2001-02) per manufacturer's specification. After 48 h transfections cells were allowed to recover for a further 24 h before processing. Non-diabetic and diabetic organ-cultured corneas were transfected with 50 nM hsa-miR-10b mimic or miR-10b-5p inhibitor, respectively, or their corresponding negative controls for 48 h using Lipofectamine RNAiMAX in submerged culture, followed by a further 72-h recovery at the air-liquid interface before being processed.

2.5.Total RNA Isolation, Library Preparation and Next generation RNA Sequencing.

2.5.1. Total RNA Isolation

The concentration and quality of the total RNA were evaluated using a NanoDrop 8000 spectrophotometer (Thermo Fisher), Qubit 2.0 Fluorometer (Thermo Fisher), and Agilent 2100 Bioanalyzer (Agilent Technologies, Santa Clara, CA, USA).

2.5.2 Library Preparation and RNA Sequencing

Total RNA samples were analyzed for RNA integrity on the 2100 Bioanalyzer using the Agilent RNA 6000 Nano Kit (Agilent Technologies) and quantified using the Qubit RNA HS Assay Kit (Thermo Fisher). An input up to 10 ng RNA was used for oligo(dT) primed reverse transcription, followed by cDNA amplification and cleanup. Quantification of cDNA was performed using Qubit (Thermo Fisher). cDNA normalized to 80 pg/ml was fragmented, and

sequencing primers were added simultaneously. A limiting-cycle PCR added Index 1 (i7) adapters, Index 2 (i5) adapters, and limiting-cycle PCR added Index 1 (i7) adapters, Index 2 (i5) adapters, and sequences required for cluster formation on the sequencing flow cell. Indexed libraries were then cleaned, library concentration was measured with a Qubit fluorometer (Thermo Fisher), and library size was evaluated on a 4200 TapeStation (Agilent Technologies). Multiplexed libraries were sequenced on a NovaSeq 6000 (Illumina San Diego, CA, USA) using 75 bp single-end sequencing. On average, approximately 30 million reads were generated from each sample.

2.5.3 RNA sequencing Data Analysis

Raw sequencing data was demultiplexed and converted to fastq format using bcl2fastq v2.20 (Illumina). Reads were aligned to the transcriptome using STAR (version 2.6.1) (5) / RSEM (version 1.2.28) (6) with default parameters, using a custom human GRCh38 transcriptome reference downloaded from http://www.gencodegenes.org, containing all protein coding and long non-coding RNA genes based on human GENCODE version 24 annotation. DESeq2 (version 1.46.0) (7) was used for normalization and principal component analysis. Batch effect was corrected using limma (8). Visualization of significant DE genes, including heatmaps and volcano plots, was performed using the ggplot2 package (v3.0.3) (9) in R. DEGs were further analyzed for KEGG and Gene Ontology (GO) enrichment using cluster Profiler (v3.20) (10).

2.6.Protein Extraction and Liquid Chromatography-Mass Spectrometry (LC-MS/MS) Analysis and Data Acquisition and Analysis

2.6.1 Protein Extraction and Liquid Chromatography-Mass Spectrometry (LC-MS/MS) Analysis

Primary LECs isolated from four individual human corneas (n = 6) were transfected with miR-10b and its respective control (miR-MC) for 48 h and allowed to recover for a further 24 h in cell culture media before processing for the respective studies. The cells were lyophilized for proteomics analysis. Lyophilized samples were suspended in 30ul of 6M urea, 0.1% RapiGest SF surfactant (Waters, Cat #186008740) Tris, sonicated with the QSonica Q800R3 Sonicator

for 10 min, using a 10 second on/off pulse. BCA assay was performed and an aliquot of 50 µg of total protein was processed using sequential incubations with 10mM Dithiothreitol for 30 minutes at 37℃ and 100mM iodoacetamide for 30 minutes at room temperature in the dark to reduce and alkylate cysteine residues. Sample pH and volume was adjusted to 8 with 200mM ammonium bicarbonate to dilute urea to 2M; acetonitrile was added to a final concentration of 10% and sequencing grade modified trypsin (Promega, Cat#V5111) was added at a ratio of 1:40 total protein. Samples were digested overnight with agitation at 37℃. Digested peptides were desalted on NEST C18 tips (Fisher Scientific, Cat #NC0194358) and dried under vacuum to remove organic solvents. Dried peptides were resuspended to 1ug/ul in a solution of 0.1% formic acid in double-distilled water prior to acquisition. (11,12)

2.6.2 Proteomics Data Acquisition and Analysis

Mass spectrometry data were acquired on Fusion Lumos Orbitrap (Thermo Fisher) instrument. Desalted peptides were separated on an Ultimate 3000 liquid chromatography system with a 60-min gradient. Peptides were separated on a preformed gradient (ranging from 0-45% organic phase) on a Pharmafluidics capLC column (Thermo Fisher) at a flow rate of 9.5 µl/min. Source parameters included spray voltage at 3.9 kV, ion transfer temp of 290°C. MS1 resolution was set to 120,000 and AGC was set to 600,000 (150% normalized AGC target) with maximum injection time of 50 ms, RF lens % was 30. Mass range of 400-1000 and AGC target value for fragment spectra of 400% were used. Peptide ions were fragmented using HCD at a normalized collision energy of 30%. Fragmented ions were detected across 40 DIA windows of 15 Da. MS2 resolutions was set to 15,000 with max injection time of 30 ms. All data was acquired in profile mode using positive polarity. A sample specific library was generated using DIA-Umpire (13,14) based signal extraction followed by matching of DIA-Umpire pseudospectra (from Q1 files only) using the Trans Proteomic Pipeline (TPP, v5.2.0) spectral matching algorithms Comet (15) and X!Tandem (16). Peptide level target-decoy probability scoring was performed by peptide prophet in the TPP (17), run individually on each search algorithm run and then results of multiple searches were combined using the TPP InterProphetParser. Peptides with probability >0.95 were compiled into a preliminary library using TPP SpectraST and retention times were aligned to iRT using Biognosys iRT standard

peptides (Biognosys, Schlieren, Switzerland). iRT aligned libraries were consolidated and converted to TraML format and randomized decoy sequences were appended. The sample specific library was then searched against each individual DIA file using openSWATH peak picking and scoring algorithm (18). Decoy-target probability modeling was done using pyProphet algorithm (19) and results from individual files were aligned across the experiment using the TRIC workflow (20). Following normalization to total MS2 signal, mapDIA (PMID: 26381204) was used to perform protein abundance inference and statistical comparisons. Proteomics data analysis was conducted using ProteoDA (v1.0.1) (21). Only prototypic peptides, peptides which can only be assigned to a single protein, were used for quantification, and proteins had to be identified by at least two prototypic peptides. The data were log2transformed, with treatment groups miR-10b (M), and control (miR-MC) and batch information included as covariates in the model design. A linear model was applied to identify proteins with differential expression (p-value < 0.05). clusterProfiler (v3.20) (10) was used to perform KEGG and Gene Ontology (GO) enrichment analyses on the differentially expressed proteins. Mass spectrometry data is uploaded at MassIVE (ftp://MSV000097328@massive.ucsd.edu) (ID: MSV000097328 or PRIDE ID: PXD061853).

2.7 Immunostaining and Western blot Analysis

2.7.1 Immunostaining

Cultured primary LECs or 5-μm thick transverse corneal cryostat sections were fixed using 10% formalin and 1% formalin, respectively. After fixation, slides are washed four times with PBS for 15 min. Cultured cells were permeabilized after washing with 0.5% Triton X-100 in PBS at room temperature for 10 min and then blocked for 1 h in 5% bovine serum albumin (BSA) in PBS. Both the cultured cells and corneal tissues were incubated with primary antibodies (Supplementary Table S2) overnight at 4°C. This was followed the next day by a series of five 5-minute washes and 1 h at room temperature incubation with cross-species adsorbed secondary antibodies conjugated with either fluorescein isothiocyanate (FITC) or tetramethyl rhodamine (TRITC) (Jackson ImmunoResearch Laboratories, West Grove, PA, USA). After application of the secondary antibodies the slides were washed in PBS three times

for 10 min. The slides were subsequently mounted with ProLong Gold Antifade Mountant containing DAPI (Thermo Fisher, cat# P36931). When capturing images, the same exposure time was used for each marker, across conditions. The images provided are representative of two to three independent experiments. As negative controls, samples without a primary antibody were included in every experiment. Antibodies for tissue sections were diluted in PBS whereas antibodies used for cultured cells were diluted in a PBS containing 10% normal goat serum and 0.5% Triton X-100.

2.7.2 Western blot analysis

Human primary LECs were transfected with an hsa-miR-10b or miR-MC for 48 h, and after a 24-hour recovery period, the cells were lysed using RIPA Lysis and Extraction Buffer (Pierce, Thermo Fisher, cat# 89900) supplemented with a protease inhibitor EDTA-free cocktail. The lysates were centrifuged at 14,000 rpm for 15 minutes at 4°C. The resulting supernatants were collected for subsequent analysis. For HCEC cells, following the recovery period, the cells were starved for 3 h in EpiLife medium (Thermo Fisher, cat# MEPI500CA) containing 1X antibiotic/antimycotic (Thermo Fisher, cat #15240062). Post-starvation, the cells were treated with 200 μM hydrogen peroxide (Fisher Science Education, cat# S25360) for 3, 6, 9, and 24 h. At each time point, lysates were collected using the same lysis protocol as for the LECs. Equal amounts of protein lysates were separated via SDS-PAGE on 4%–20% gradient Tris-glycine SDS polyacrylamide gels (Thermo Fisher, cat# XP04205BOX) and transferred to nitrocellulose membranes in Tris-Glycine buffer system. The membranes were blocked with 5% non-fat dry milk (Bio-Rad, Hercules, CA, USA, cat# 1706404XTU) for 1 h at room temperature and then incubated overnight at 4°C with primary antibodies (Supplementary Table S2). IRDye 800CW or 680RD goat anti-mouse or anti-rabbit secondary antibodies (LI-COR Biosciences, Lincoln, NE, USA) were used for detection. The blots were visualized and quantified using Image Studio software within Odyssey CLX imaging system (LI-COR Biosciences). The target protein band intensities were normalized to β-actin levels.

2.8 GSH-Glo™ Glutathione Assay

Human primary LECs were seeded into 96-well white opaque plates at a density of 2,500 cells per well in the specified growth medium and transfected with the hsa-miR-10b, miR-MC, siRNA-GCLM, and its negative control for 48 h and allowed to recover for a further 24 h as described above. Subsequently, after 3 h starvation period the cells were treated with 200 μM hydrogen peroxide for 6 h. The cells were maintained in an incubator at 37°C with 5% CO_2 throughout the procedure. Before starting the assay, the growth medium was carefully aspirated to avoid disrupting the cell monolayer. Glutathione levels were quantified using the GSH Glo™ Glutathione Assay (Promega Corporation, WI, USA, cat# V6611) with reagents freshly prepared according to the manufacturer's protocol. Specifically, the GSH-Glo™ Reagent was formulated by combining the Luciferin-NT substrate and Glutathione S-Transferase (GST) with the GSHGlo™ Reaction Buffer in a 1:100 ratio. A volume of 100 μL of the prepared reagent was added to each well, and the plate was gently agitated on a plate shaker for 30 seconds to ensure even distribution. The reaction was incubated at room temperature for 30 min in the dark, after which 100 μL of the reconstituted Luciferin Detection Reagent was added to each well. The plate was again briefly shaken and incubated for an additional 15 min at room temperature. Luminescence was measured using LUMIstar Omega luminescence plate reader with an integration time of 0.25 to 1 sec per well. To calculate absolute glutathione concentrations, luminescence values were normalized to negative controls, and net luminescent signals were determined by subtracting background readings. A standard curve was generated using serial dilutions of a known glutathione standard. The assay was performed in triplicate for each donor, with replicates for each condition, and the results were analyzed using GraphPad Prism (Version 9.2.0). Data are presented as mean ± standard deviation.

2.9 DCFDA/H2DCFDA-Cellular ROS Assay

HCEC were then transfected with hsa-miR-10b or miR-MC, as mentioned before. Following an initial 3-hour starvation period, LECs were then treated with 200 μM hydrogen peroxide and evaluated after 3, 6, 9 and 24 h. Reactive oxygen species were measured using the DCFDA / H2DCFDA - Cellular ROS Assay Kit (Abcam, Waltham, MA, cat# ab113851), with preparation of reagents following manufacturers protocol. Microplate assay (LECs in 96-well

plate) and fluorescent microscopy measurement (LECs in 48-well plate) were conducted to quantify and visually assess ROS activity. After 24 h recovery from transfection, cell media was removed, and cells were washed with 1X buffer. To serve as a positive control, 55 μM of prepared TBHP solution diluted in 1X supplemented buffer was added to the wells, 4 h prior to the testing point. HCECs and their positive controls were then stained with a 20 μM solution of DCFDA diluted in 1X buffer and subsequently incubated for 45 min at 37°C in the dark. Readings of fluorescence for ROS activity were conducted using fluorescence plate readers at Ex/Em = 485/535 nm. HCECs on the 48-well plate were stained with DCFDA solution and incubated, following protocols similar to the microplate assay. Live cell images were taken with a confocal microscope (Revolve, Echo Laboratories) using the FITC filter.

Data and Resource Availability

Transcriptomics data: To review GEO accession GSE292721: Go to

https://urldefense.com/v3/__https://www.ncbi.nlm.nih.gov/geo/query/acc.cgi?acc=GSE292721__;!!KOmnBZxC8_2BBQ!yywj0Zs8BFDf0IYCva0HYzzMtom6UBgNJ3oRKSjbOQeCw7vXPUU6lT8V3psrbs-b59xqXIV7UYDmFsfUDjxi$

Proteomics data: Mass spectrometry data is uploaded at MassIVE (ftp://MSV000097328@massive.ucsd.edu) (ID: MSV000097328 or PRIDE ID: PXD061853).

Supplemental references

1. Winkler MA, Dib C, Ljubimov AV, Saghizadeh M. Targeting miR-146a to treat delayed wound healing in human diabetic organ-cultured corneas. PLoS One 2014;9:e114692.
2. Blazejewska EA, Schlötzer-Schrehardt U, Zenkel M, Bachmann B, Chankiewitz E,Jacobi C, et al. Corneal limbal microenvironment can induce transdifferentiation of hair follicle stem cells into corneal epithelial-like cells. Stem Cells 2009;27:642–652.
3. Dimitrijevich SD, Shankardas J. Characterization of telomerized human corneal epithelial cell line. Invest Ophthalmol Vis Sci 2008;49:4306.

4. Kabosova A, Kramerov AA, Aoki AM, Murphy G, Zieske JD, Ljubimov AV. Human diabetic corneas preserve wound healing, basement membrane, integrin and MMP-10 differences from normal corneas in organ culture. Exp Eye Res 2003;77:211–217.
5. Dobin A, Davis CA, Schlesinger F, Drenkow J, Zaleski C, Jha S, et al. STAR: ultrafast universal RNA-seq aligner. Bioinformatics 2013;29:15–21.
6. Li B, Dewey CN. RSEM: accurate transcript quantification from RNA-seq data with or without a reference genome. BMC Bioinformatics 2011;12:323.
7. Love MI, Huber W, Anders S. Moderated estimation of fold change and dispersion for RNAseq data with DESeq2. Genome Biol 2014;15:550.
8. Ritchie ME, Phipson B, Wu DI, Hu Y, Law CW, Shi W, et al. limma powers differential expression analyses for RNA-sequencing and microarray studies. Nucleic Acids Res 2015;43:e47.
9. Wickham H, Sievert C. ggplot2: elegant graphics for data analysis. Springer; 2009.
10. Yu G, Wang LG, Han Y, He QY. clusterProfiler: an R package for comparing biological themes among gene clusters. Omics 2012;16:284–287.
11. Stotland AB, Spivia W, Orosco A, et al., (2020). MitoPlex: A targeted multiple reaction monitoring assay for quantification of a curated set of mitochondrial proteins 2020; 142:1–13.
12. Robinson AE, Binek A, Venkatraman V, et al., Lysine and arginine protein post-translational modifications by enhanced DIA libraries: Quantification in murine liver disease. J Proteome Res 2020;19:4163−4178.
13. Zhang F, Ge W, Ruan G, Cai X, Guo T. Data-independent acquisition mass spectrometrybased proteomics and software tools: a glimpse. Proteomics 2020;20:e1900276.
14. Tsou CC, Avtonomov D, Larsen B, Tucholska M, Choi H, Gingras AC, et al. DIA-Umpire: comprehensive computational framework for data-independent acquisition proteomics. Nat Methods 2015;12:258–264.
15. Eng JK, Jahan TA, Hoopmann MR. Comet: an open-source MS/MS sequence database search tool. Proteomics 2013;13:22–24.
16. Keller A, Nesvizhskii AI, Kolker E, Aebersold R. Empirical statistical model to estimate the accuracy of peptide identifications made by MS/MS and database search. Anal Chem 2002;74:5383–5392.

17. Deutsch EW, Mendoza L, Shteynberg D, Slagel J, Sun Z, Moritz RL. Trans-Proteomic Pipeline, a standardized data processing pipeline for large-scale reproducible proteomics informatics. Proteomics Clin Appl 2015;9:745–754.
18. Röst HL, Rosenberger G, Navarro P, Gillet L, Miladinović SM, Schubert OT, et al. OpenSWATH enables automated, targeted analysis of data-independent acquisition MS data. Nat Biotechnol 2014;32:219–223.
19. Reiter L, Rinner O, Picotti P, Hüttenhain R, Beck M, Brusniak MO, et al. mProphet: automated data processing and statistical validation for large-scale SRM experiments. Nat Methods 2011;8:430–435.
20. Röst HL, Liu Y, D'Agostino G, Zanella M, Navarro P, Rosenberger G, et al. TRIC: an automated alignment strategy for reproducible protein quantification in targeted proteomics. Nat Methods 2016;13:777–783.
21. Kim SY, Parker JK, Gonzalez-Magaldi M, Telford MS, Leahy DJ, Davies BW. Export of diverse and bioactive peptides through a type I secretion system. bioRxiv 2023.

Supplementary Information

Supplementary Table S1: Donor characteristics

Case number	Age	Sex	Race	Cause of death	Types	DM duration, years
N 13-34	79	F	Caucasian	Hip fracture	N/A	N/A
N 17-04	75	M	Caucasian	Cardiac arrest	N/A	N/A
N 17-11	52	M	Caucasian	Hypertension	N/A	N/A
N 18-19	73	M	Caucasian	Multi-organ failure	N/A	N/A
N 18-23	57	F	Caucasian	CVA / Stroke	N/A	N/A
N 19-04	71	M	Caucasian	Cardiac arrest	N/A	N/A
N 19-06	59	F	Caucasian	Peritoneal carcinoma	N/A	N/A
N 19-13	66	M	Caucasian	Cardiac arrest	N/A	N/A
N 19-32	70	M	Caucasian	Respiratory failure	N/A	N/A
N 19-35	35	M	Caucasian	KCl Overdose	N/A	N/A
N 20-06	63	M	Caucasian	Cardiac arrest	N/A	N/A
N 20-14	80	F	Caucasian	Cardiac arrest	N/A	N/A
N 20-26	75	F	Caucasian	Cardiac arrest	N/A	N/A
N 23-03	80	M	Caucasian	Cardiac arrest	N/A	N/A
N 23-04	34	M	Caucasian	Choking / Drug Overdose	N/A	N/A
N 23-09	73	F	Caucasian	Chronic Obstructive Pulmonary Disease	N/A	N/A
N 23-13	62	M	Caucasian	Myocardial infarction	N/A	N/A
N 23-15	77	F	Caucasian	Intracranial hemorrhage	N/A	N/A
N 23-21	74	M	Caucasian	Pneumonia	N/A	N/A
N 24-07	55	M	Caucasian	CVA / Stroke	N/A	N/A
N 24-24	54	M	Caucasian	Myocardial infarction	N/A	N/A
DM 22-20	73	F	Caucasian	Anoxia	II	34
DM 22-21	73	M	Caucasian	Anoxia	II	10
DM 22-28	76	M	Caucasian	Cardiac arrest	II	30
DM 23-19	64	F	Caucasian	Anoxia brain injury	II	20
DM 23-25	60	F	Caucasian	End stage renal disease	II	30
DM 23-32	75	M	Caucasian	CVA / Stroke	II	25
DM 23-34	69	M	Caucasian	End stage renal disease	I	20
DM 24-11	70	M	Hispanic	End stage renal disease	II	25-30
DM 24-18	70	F	Caucasian	Myocardial infarction	II	Unknown
DM 24-29	64	F	Asian	Anoxia	II	20

Supplementary Table S2: List of used antibodies

Antigen	Antibody	Source	Assay	Dilution
β-Actin	Mouse mAb 3700	Cell Signaling Technology	WB	1:1000
GCLC	Rabbit mAb ab207777	Abcam	WB	1:2000
GCLM	Rabbit mAb ab126704	Abcam	WB, IHC	1:1000, 1:100
LANCL1	Rabbit pAb ab234827	Abcam	WB,IHC	1:1500,1:100

mAb, monoclonal antibody; pAb, polyclonal antibody; WB, western blot; IHC, immunohistochemistry
